# Systolic and diastolic blood pressure, prostate cancer risk, treatment, and survival. The PROCA‐*life* study

**DOI:** 10.1002/cam4.4523

**Published:** 2021-12-22

**Authors:** Einar Stikbakke, Henrik Schirmer, Tore Knutsen, Martin Støyten, Tom Wilsgaard, Edward L. Giovannucci, Anne McTiernan, Anne E. Eggen, Hege S. Haugnes, Elin Richardsen, Inger Thune

**Affiliations:** ^1^ Department of Clinical Medicine Faculty of Health Sciences UiT The Arctic University of Norway Tromsø Norway; ^2^ Department of Oncology University Hospital of North Norway Tromsø Norway; ^3^ Department of Cardiology Akershus University Hospital Lørenskog Norway; ^4^ Institute of Clinical Medicine Campus Ahus University of Oslo Oslo Norway; ^5^ Department of Urology University Hospital of North Norway Tromsø Norway; ^6^ Department of Community Medicine Faculty of Health Sciences UiT The Arctic University of Norway Tromsø Norway; ^7^ Department of Medicine Brigham and Women’s Hospital and Harvard Medical School Boston Massachusetts USA; ^8^ Departments of Nutrition and Epidemiology Harvard T.H. Chan School of Public Health Boston Massachusetts USA; ^9^ Program in Epidemiology Division of Public Health Sciences Fred Hutchinson Cancer Research Center Seattle Washington USA; ^10^ Department of Epidemiology School of Public Health, and Department of Medicine, School of Medicine University of Washington Seattle Washington USA; ^11^ Department of Medical Biology Faculty of Health Sciences UiT The Arctic University of Norway Tromsø Norway; ^12^ Department of Pathology University Hospital of North Norway Tromsø Norway; ^13^ Institute of Clinical Medicine Faculty of Medicine University of Oslo Oslo Norway; ^14^ Department of Oncology, The Cancer Centre, Ullevaal Oslo University Hospital Oslo Norway

**Keywords:** hypertension, inflammation, mortality, prostate cancer, risk

## Abstract

**Background:**

Inflammation has been linked to prostate cancer and hypertension, but it remains equivocal whether elevated blood pressure (BP) influence prostate cancer risk and survival.

**Method:**

Using Cox regression models, we examined the association between prediagnostic BP and prostate cancer risk among 12,271 men participating in the Prostate Cancer throughout life (PROCA‐life) study. Systolic and diastolic BP were measured. A total of 811 men developed prostate cancer, and followed for additional 7.1 years, and we studied the association between prediagnostic BP and overall mortality among patients with prostate cancer.

**Results:**

Men (>45 years) with a systolic BP >150 mmHg had a 35% increased risk of prostate cancer compared with men with a normal systolic BP (<130 mmHg) (HR 1.35, 95% CI 1.08–1.69). Among patients with prostate cancer, men with systolic BP >150 mmHg had a 49% increased overall mortality compared with men with a normal systolic BP (HR 1.49, 1.06–2.01). Among patients with prostate cancer treated with curative intent, those with a high diastolic BP (>90 mmHg) had a threefold increase in overall mortality risk (HR 3.01, 95% CI 1.40–6.46) compared with patients with a normal diastolic BP (<80 mmHg).

**Conclusion:**

Our results support that systolic and diastolic BP are important factors when balancing disease management in patients with prostate cancer.

## Graphical abstract

Systolic and diastolic blood pressure were measured in 12 271 men in a population based cohort, and during a mean of 20.0 years of follow‐up, a total of 811 men developed prostate cancer. A dose response association was observed between prediagnostic systolic and diastolic BP and prostate cancer risk and overall survival. These findings underline that both systolic and diastolic BP are important factors in prostate cancer patients.

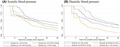

## 1 INTRODUCTION

Prostate cancer and hypertension are both common and complex conditions among men world‐wide. While prostate cancer is one of the most common cancers in men and its incidence continues to rise, systolic blood pressure (BP) above 115 mmHg is ranked as a leading risk factor for the global burden of disease.^1^ The global age‐standardized prevalence of elevated BP (systolic BP ≥140 mmHg or diastolic BP ≥90 mmHg) in men was estimated as ≥20% in 2015.^2^ Of note, high BP may last for several decades ahead of any disease development, reflecting a long‐lasting cumulative exposure and exposure time of interest in an ageing‐related disease as prostate cancer.

Hypertension has been linked to inflammation, and inflammation is one of the hallmarks of cancer development.^3^ Inflammatory cells in the prostate microenvironment associated with precursor lesions for prostate cancer in the prostate gland, termed proliferative inflammatory atrophy, have been observed.^4^ Recently, we observed that systemic prediagnostic inflammatory biomarkers including high sensitive C‐reactive protein (hs‐CRP) and white blood cells were associated with prostate cancer development, and our results are supported by others linking systemic inflammatory biomarkers to prostate cancer development.^5^

Results from previous studies of the association between hypertension and prostate cancer development have been inconsistent.^6^, ^7^, ^8^ Neither the European Prospective Investigation into Cancer and Nutrition (EPIC) nor a meta‐analysis observed any association between hypertension and risk of prostate cancer.^7^, ^8^ However, a meta‐analysis including case–control and cohort studies support that hypertension may increase prostate cancer risk.^6^ Moreover, in a longitudinal case–control study, men (aged 40–58 years at study entry) in the highest quartile of systolic BP (>150 mmHg) had an increased prostate cancer risk.^9^ Hypertension was also associated with increased risk of biochemical recurrence after radical prostatectomy, independent of age at diagnosis and tumor pathological features.^10^ Whether long‐lasting, raised diastolic hypertension influences prostate cancer development and prognosis has not been much studied. Use of antihypertensive medication does not seem to have any effect on cancer risk.^11^ Thus, the importance of elevated BP may show variation by age at onset of hypertension, exposure time, age when diagnosed with prostate cancer, and aggressiveness of disease.^12^

Whether long‐lasting, modern, prostate cancer treatments interact with systolic and diastolic BP of importance for survival has not been much studied.^13^ Androgen deprivation therapy (ADT) has a key role in adjuvant prostate cancer treatment combined with radiation therapy, as well as in the lifelong treatment of metastatic prostate cancer.^14^, ^15^ However, important side effects from ADT include a higher risk of later cardiovascular disease (CVD).^16^ Men with prostate cancer, aged ≥40 years, who underwent ADT, were observed to have a higher risk of developing hypertension.^17^ However, there is a knowledge gap regarding elevated BP before, during, and after prostate cancer treatment. Furthermore, we lack information about the importance of a pre‐existing hypertension on the risk for future CVD events after initiating ADT among patients with prostate cancer.

The aim of the present study was, therefore, to study whether prediagnostic systolic and diastolic BP were associated with prostate cancer risk, if prediagnostic systolic and diastolic BP were associated with overall mortality among patients with prostate cancer, and if such associations vary by age and type of prostate cancer treatment.

## 2 METHOD

### 2.1 Study design, settings, and participants

The Prostate Cancer Study throughout life (PROCA‐life) includes all men older than 25 years at study entry who were enrolled in the population‐based Tromsø Study in 1994/1995 (Tromsø 4).^18^, ^19^ The procedures and assessments were performed by trained research technicians at one study site. All age‐eligible men in the Tromsø municipality were invited to participate with a personal written invitation, and nonresponders were given one reminder. The attendance proportion for men included in the present study was 69.6% of those invited.^19^

### 2.2 Questionnaire and assessments of lifestyle factors

The questionnaire was checked for completeness and inconsistency and included questions about medical history, lifestyle factors, and use of medication including antihypertensive drugs. Educational level was categorical (1 = secondary school only, 5 = college/university for 4 or more years). Alcohol use was defined as more than 1 unit of alcohol per month, defined by others in this cohort.^20^, ^21^ We defined being physically active as more than 1 h/week of strenuous exercise, or any leisure time exercise more than 2–3 times/week.

### 2.3 Assessments of systolic and diastolic blood pressure and clinical assessments

Systolic and diastolic BP (mmHg) were measured by using an automatic device (Dinamap Vital Signs Monitor 1846; Critikon Inc.). Participants rested for 2 min in a sitting position, then three readings were taken on the upper right arm, separated by 1‐min intervals, and the average of the last two readings was used.^22^

Height and weight were measured on a regularly calibrated electronic scale with the participants wearing light clothing and no shoes. Height was measured to the nearest centimeter (cm) and weight to the nearest kilogram (kg). Body mass index (BMI) was calculated using the formula weight/height^2^ (kg/m^2^).

### 2.4 Assessment of serum samples

Blood samples (nonfasting) were drawn by trained research assistants on attendance. Analyses of serum samples were done at the Department of Laboratory Medicine, University Hospital of Northern Norway (UNN).^18^ Prostate‐specific antigen (PSA) measurements were done for cancer cases only, as part of clinical routine in diagnosis and follow‐up (1990–1994 Stratus^®^ PSA Fluorometric Enzyme Immunoassay, 1994–2001 AxSYM Psa Reagent Pack, Abbot^®^, 2001 Bayer^®^ PSA Reagens Pack Immuno I [Prod. Nr.T01‐3450‐51], Technicon Immuno I). For patients with prostate cancer diagnosed or treated in other institutions (*n* = 21), PSA values from their local laboratories were recorded.

### 2.5 Identification of patients with prostate cancer during follow‐up

Patients with prostate cancer diagnosed during follow‐up (until December 31, 2018) were identified by using the unique national 11‐digit identification number through linkage with the Cancer Registry of Norway. We excluded all men who had a previous history of cancer (*n* = 382), or who emigrated, died, or were diagnosed with cancer within the first year after study entry (*n* = 128), to account for the possibility that undiagnosed cancer or severe illness could influence our results. Participants with missing measurement of BP at study entry were also excluded (*n* = 24) leaving a final study population of 12,271 men (Figure S1).

A total of 811 men developed prostate cancer during follow‐up between 1994 and 2018. There were no ongoing screening programs for prostate cancer in Norway during the study period. Causes of death were identified by linkage to the Norwegian Cause of Death Registry, and dates of emigration were obtained from the Population Registry of Norway.

Detailed information from medical records were obtained by trained physicians (TK, MS, and ES) and included prostate cancer treatments and recurrence. A total of 153 patients with prostate cancer had missing data in treatment details or follow‐up but were still included if baseline data; data about diagnosis and data on cause of death were complete (Figure S1).

Histopathological information for the patients with prostate cancer was obtained from histopathological records and were in addition re‐examined by the same specialized pathologist (ER) and classified according to the latest International Society of Urological Pathology (ISUP) guidelines on Gleason score and ISUP grade group.^23^ Patients with prostate cancer were divided into four risk groups based on PSA level at diagnosis, highest ISUP grade group and clinical T‐stage, similar to the European Association of Urology‐classification (EAU) guidelines.^14^ Risk group 1 (low) was defined as PSA <10 µg/L, clinical T‐stage (cT‐) 1, and ISUP grade group 1. Risk group 2 (intermediate) was defined as PSA: 10–20 µg/L, cT‐stage 2, or ISUP grade group 2–3. Risk group 3 (high) was defined as PSA: >20–100 µg/L, cT‐stage 3, or ISUP grade group 4–5. Risk group 4 (metastatic) was defined as PSA >100 µg/L, or with radiological evidence of metastatic disease. ISUP grade group was reported after reclassification when available. PSA values above 100 were not included in calculation of mean or median PSA.

### 2.6 Statistical methods

Descriptive characteristics of the study population were presented as means (standard deviation) or percent (numbers). Multivariable Cox proportional hazard models, with follow‐up time as timescale, were used to investigate whether prediagnostic systolic or diastolic BP were independently associated with prostate cancer risk and mortality. To study the importance of the variation, prediagnostic systolic and diastolic BP were split in four levels based on international categories: systolic BP (mmHg): <130, 130–139.9, 140–149.9, ≥150 mmHg, diastolic BP (mmHg): <80, 80–89.9, 90–99.9, ≥100 mmHg.

Associations between baseline BP and prostate cancer incidence have been studied in the full cohort (*n* = 12,271), and associations between baseline BP and overall mortality have been studied in men diagnosed with prostate cancer (the PCa‐cohort, *n* = 811). Follow‐up to incidence of prostate cancer was calculated from the date of entry into the study to the date of prostate cancer diagnosis, date of emigration, date of death, or end of follow‐up (December 31, 2018), whichever event occurred first. Follow‐up to mortality after prostate cancer diagnosis was calculated from the date of prostate cancer diagnosis to date of death, emigration, or end of follow‐up (December 31, 2018). Based on biological mechanisms hypothesized and previous observations suggesting that risk factors for prostate cancer may vary by time period during lifetime and by length of exposure,^24^ separate analyses on prostate cancer incidence were performed in two age groups (age at entry <45 years and age >45 years). Furthermore, to study whether the association between prediagnostic BP and mortality varied by the type of prostate cancer treatment, analyses were performed by type of treatment, curative or endocrine, within the PCa‐cohort.

Several variables were assessed as potential confounders based on suggested biological mechanisms influencing systolic and diastolic BP and/or prostate cancer risk and prognosis. Age at entry (continuous), BMI (continuous), alcohol habits (categorical), smoking (categorical), physical activity (categorical), educational level (categorical), and diabetes (yes/no) were included as covariates in the final models. Use of lipid‐lowering and/or antihypertensive medication were included but did not influence the results and were excluded in the final models.

Kaplan–Meier survival curves of prostate cancer incidence and of total mortality were presented for the full cohort and for the PCa cohort, respectively. The proportional hazard assumption was verified by assessing the parallelism between log minus log survival curves for categories of BP and also formal tests based on Schoenfeld residuals. All statistical tests were two‐sided using a significance level of *p* < 0.05 and conducted with STATA/MP version 16 (StataCorp LLC).

## 3 RESULTS

At study entry, the cohort participants had the following means: age at entry 45.6 years (SD 14.2), prediagnostic systolic BP 134.1 mmHg and prediagnostic diastolic BP 77.5 mmHg (Table 1). During follow‐up, a total of 811 men developed prostate cancer with a mean age at diagnosis of 69.4 years. A total of 18.0% of the patients with prostate cancer were in the low‐risk group, and 21.7% were in the high‐risk group at the time of diagnosis. A total of 265 patients with prostate cancer (32.7%) died during 7.1 years of follow‐up, of whom 41.9% (*n* = 111) were classified as prostate cancer death, 12.5% (*n* = 33) as cardiovascular death and 45.7% (*n* = 121) other causes of death (Table 1, Table S2).

**TABLE 1:** Distribution of selected prediagnostic characteristics for men with prostate cancer (cases) and without prostate cancer (non‐cases) in the PROCA‐life Study (1994–2018)

Characteristics	Non‐cases (*n* = 11,460)	Prostate cancer cases (*n* = 811)	
Age at entry (years)	45.6 (14.2)	54.4 (10.8)	
Observation time (years)	21.0 (6.0)	14.0 (6.1)	
Clinical variables, mean (SD)			
Systolic blood pressure (mmHg)	134.1 (16.8)	137.9 (18.9)	
Diastolic blood pressure (mmHg)	77.5 (11.6)	80.8 (11.7)	
Body mass index (kg/m^2^)	25.6 (3.3)	25.9 (3.2)	
Serum samples at study entry mean (SD)			
Total cholesterol (mmol/L)	6.02 (1.2)	6.32 (1.2)	
Hs‐CRP (mg/L)^a^	2.97 (7.4)	2.57 (4.7)	
White blood cells (×10^9^/L)	7.07 (2.0)	6.98 (1.8)	
Lifestyle factors (%)			
Lipid‐lowering drugs, current use	1.0	1.4	
User of blood pressure–lowering medication	7.2	9.3	
Current smokers	36.8	31.0	
Physically active	37.6	36.0	
Alcohol user	66.5	66.8	
Characteristics among patients with prostate cancer			
Age at diagnosis, mean (SD) (years)		69.4 (9.0)	
PSA at diagnosis, median (μg/L)^b^		10.9	
Observation time after diagnosis (years)		7.1	
Cancer‐specific mortality, % of all death (*n*)		41.9 (111)	
Cardiovascular death, % of all death (*n*)		12.5 (33)	
Other causes, % of all death (*n*)		45.7 (121)	
Tumor characteristics			
T‐stage, % (*n*)			
T1		42.4 (344)	
T2		24.4 (198)	
T3		13.1 (106)	
T4		3.8 (31)	
Tx		16.2 (132)	
ISUP Grade Group, % (*n*)			
1 (Gleason 3+3)		39.1 (317)	
2 (Gleason 3+4)		19.5 (158)	
3 (Gleason 4+3)		8.5 (69)	
4 (Gleason 4+4)		6.9 (56)	
5 (Gleason 4+5/5+4/5+5)		7.4 (60)	
ISUP missing		16.8 (151)	
Risk group, % (*n*)			
Low		18.0 (146)	
Intermediate		32.9 (267)	
High		21.7 (176)	
Metastatic		9.0 (73)	
Unknown		18.4 (149)	
Prostate cancer treatment characteristics, % (*n*)			
Curative intended treatment		58.7 (476)	
Endocrine treatment, overall		36.0 (292)	
Endocrine treatment, curative		19.2 (156)

Numbers may vary due to missing information. Values are mean (standard deviation) unless otherwise specified.

Prostate cancer risk group definitions: Low: PSA <10 µg/L, clinical T‐stage (cT‐) 1, and ISUP grade group 1. Intermediate: PSA: 10–20 µg/L, cT‐stage 2, or ISUP grade group 2–3. High: PSA: >20–100 µg/L, cT‐stage 3, or ISUP grade group 4–5. Metastatic: PSA >100 µg/L, or with radiological evidence of metastatic disease.

Abbreviations: Hs‐CRP, high‐sensitivity C‐reactive protein; PSA, prostate‐specific antigen; ISUP, International Society of Urological Pathology.

^a^ CRP measured only in 2781 men.

^b^ PSA values above 100 were not included in calculation of mean or median PSA.

### 3.1 Prediagnostic systolic and diastolic blood pressure and prostate cancer risk

**FIGURE 1:**
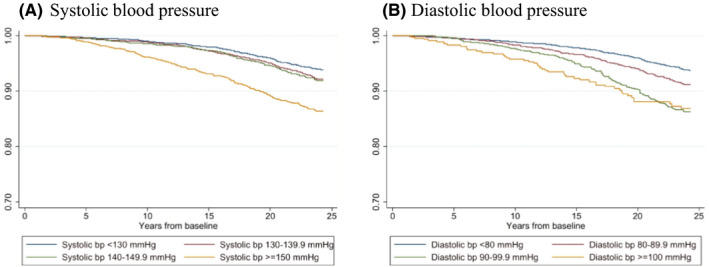
Kaplan–Meier survival curves of prostate cancer incidence according to prediagnostic systolic (A) and diastolic (B) blood pressure (bp)

We observed an increased incidence of prostate cancer among men in the upper level of both systolic and diastolic BP (systolic BP ≥150 mmHg, diastolic BP ≥100 mmHg) in crude data (Figure 1). Among men aged >45 years at study entry, we observed, when adjusted for potential confounding factors, a positive dose–response association between prediagnostic systolic BP and prostate cancer risk (HR 1.07 per SD increase, 95% CI 1.00–1.16). Furthermore, men with a prediagnostic systolic BP >150 mmHg had a 35% increased risk of prostate cancer compared with men with prediagnostic systolic BP <130 mmHg (HR 1.35, 95% CI 1.08 −1.69). We observed an overall positive dose–response relationship between prediagnostic diastolic BP and risk of prostate cancer (HR 1.08 per SD increase, 95% CI 1.01–1.17) (Table 2, Figure 1). Associations between BP and incidence of different risk‐groups of prostate cancer has been tested but did not provide statistically significant results.

**TABLE 2:** Multivariable adjusted^a^ hazard ratios (HRs) for incident prostate cancer according to the levels of prediagnostic systolic and diastolic blood pressure by age‐group (≤/>45 years). The PROCA‐*life* study (1994–2018)

	All age ( patients with prostate cancer *n* = 811)		≤45 years at baseline (patients with prostate cancer *n* = 183)		>45 years at baseline ( patients with prostate cancer *n* = 628)	
	HR (95%CI)			HR (95%CI)		HR (95%CI)
	Number of cases	Multivariable^a^	Number of cases	Multivariable^a^	Number of cases	Multivariable^a^
Systolic blood pressure (mmHg)						
<130	296	1.00 (ref.)	95	1.00 (ref.)	201	1.00 (ref.)
130–139.9	221	**1.20 (1.01–1.43)**	56	1.13 (0.81–1.58)	165	**1.28 (1.04–1.58)**
140–149.9	121	0.99 (0.80–1.23)	23	1.03 (0.65–1.64)	98	1.08 (0.84–1.38)
≥150	173	1.13 (0.92–1.39)	9	0.87 (0.43–1.74)	164	**1.35 (1.08–1.69)**
*p* for trend^b^		*0.41*		*0.967*		***0.025***
Per SD increase		1.00 (0.93–1.08)		0.94 (0.76–1.16)		**1.07 (1.00–1.16)**
Diastolic blood pressure (mmHg)						
<80	404	1.00 (ref.)	132	1.00 (ref.)	272	1.00 (ref.)
80–89.9	227	0.99 (0.83–1.16)	37	0.80 (0.55–1.15)	190	0.93 (0.77–1.13)
90–99.9	132	**1.25 (1.02–1.54)**	11	0.79 (0.42–1.49)	121	1.20 (0.96–1.50)
≥100	48	1.20 (0.88–1.64)	3	0.76 (0.24–2.40)	45	1.15 (0.83–1.59)
*p* for trend^b^		*0.056*		*0.223*		*0.165*
Per SD increase		**1.08 (1.01–1.17)**		0.88 (0.74–1.06)		1.05 (0.97–1.15)

Statistically significant (*p*‐value < 0.05) hazard ratios are marked in bold letters. *p*‐value for linear trend in blood pressure categories are marked in italic letters.

^a^ Adjusted for age at baseline, body mass index (BMI, kg/m^2^), smoking, alcohol use, physical activity, diabetes, and education level.

^b^ *p*‐value for linear trend in blood pressure categories.

### 3.2 Prediagnostic systolic and diastolic blood pressure and survival

After 7.1 years of follow‐up after being diagnosed with prostate cancer, there was among patients with prostate cancer a positive dose–response association between prediagnostic systolic BP and overall mortality (HR 1.14 per SD increase, 95% CI 1.03–1.27) and prediagnostic diastolic BP and overall mortality (HR 1.17 per SD increase, 95% CI 1.03–1.32). Patients with prostate cancer with a prediagnostic diastolic BP ≥100 mmHg, had an 85% increased overall mortality compared with patients with prostate cancer with diastolic BP <80 mmHg (HR 1.85, 95% CI 1.22–2.82). Patients with prostate cancer treated with curative intention and with a high prediagnostic diastolic BP (≥100 mmHg) had a threefold higher overall mortality risk compared with the patients with prostate cancer with a prediagnostic diastolic BP <80 mmHg (HR 3.05, 95% CI 1.42–6.55). Among patients with prostate cancer receiving endocrine treatment, those with a high prediagnostic diastolic BP (≥100 mmHg) at study entry had a twofold increase in overall mortality risk compared with those with a prediagnostic diastolic BP <80 mmHg (HR 2.15, 95% CI 1.25–3.69) (Table 3).

**TABLE 3:** Multivariable adjusted^a^ hazard ratios (HRs) for all‐cause mortality according to prediagnostic systolic and diastolic blood pressure among patients with prostate cancer by the type of treatment (curative and endocrine prostate cancer treatment). The PROCA‐*life* study (1994–2018)

		All prostate cancer		Curative treatment		Endocrine treatment
	Number of deaths/cases	265/798	Number of deaths/cases	86/476	Number of deaths/cases	168/292
		HR (95% CI)		HR (95% CI)		HR (95% CI)
Systolic blood pressure (mmHg)						
<130	67/296	1.00 (reference)	22/196	1.00 (reference)	44/94	1.00 (reference)
130–139.9	60/221	1.08 (0.75–1.55)	21/112	1.11 (0.59–2.08)	40/72	0.87 (0.55–1.36)
140–149.9	46/121	0.97 (0.65–1.47)	17/70	1.58 (0.81–3.10)	30/48	0.91 (0.55–1.51)
≥150	92/173	1.35 (0.96–1.90)	26/82	1.83 (0.99–3.40)	54/78	1.11 (0.73–1.71)
*p* for trend^b^		*0.091*		***0.029***		*0.51*
Per SD increase		**1.14 (1.03–1.27)**		**1.26 (1.03–1.55)**		1.14 (0.99–1.31)
Diastolic blood pressure (mmHg)						
<80	110/404	1.00 (reference)	32/238	1.00 (reference)	74/125	1.00 (reference)
80–89.9	75/227	1.08 (0.80–1.45)	24/134	1.10 (0.64–1.88)	48/94	0.98 (0.67–1.42)
90–99.9	50/132	1.24 (0.87–1.75)	20/80	1.75 (0.97–3.14)	26/49	0.91 (0.57–1.45)
≥100	30/48	**1.85 (1.22–2.82)**	10/24	**3.05 (1.42–6.55)**	20/24	**2.15 (1.25–3.69)**
*p* for trend^b^		***0.009***		***0.004***		*0.13*
Per SD increase		**1.17 (1.03–1.32)**		**1.43 (1.17–1.75)**		1.12 (0.97–1.30)

Statistically significant (*p*‐value < 0.05) hazard ratios are marked in bold letters. *p*‐value for linear trend in blood pressure categories are marked in italic letters.

^a^ Adjusted for age at baseline, body mass index (BMI, kg/m^2^), smoking, alcohol use, physical activity, diabetes, and education level.

^b^ *p*‐value for linear trend in blood pressure categories.

**FIGURE 2:**
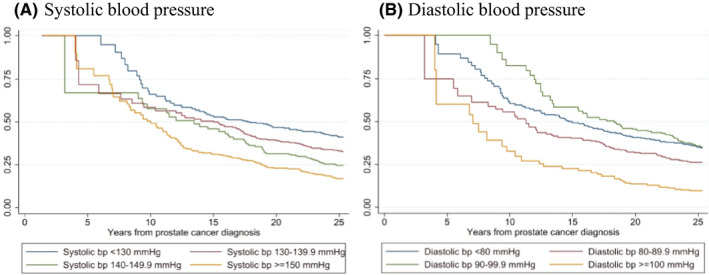
Kaplan–Meier survival curves of overall mortality among prostate cancer cases (*n* = 811) according to prediagnostic systolic (A) and diastolic (B) blood pressure (bp)

After 10 years of follow‐up, we observed that among patients with prostate cancer, 49% of those with a prediagnostic systolic BP ≥150 mmHg were alive, compared with 66% of patients with prostate cancer with a normal prediagnostic systolic BP (<130 mmHg). Among those with a prediagnostic diastolic BP ≥100 mmHg, 33% were alive, compared with 61% of the patients with prostate cancer with a normal prediagnostic diastolic BP (<80 mmHg). (Figure 2).

This association was even more pronounced among men >45 years at entry compared with overall, where the patients with prostate cancer with a high prediagnostic diastolic BP (≥100 mmHg) at study entry had a nearly doubled overall mortality risk compared with those with a prediagnostic diastolic BP <80 mmHg (HR 1.99, 95% CI 1.30–3.04), and a positive dose–response association was observed between prediagnostic BP and overall mortality (*p*‐trend = 0.002) (Table 3, Table S1).

## 4 DISCUSSION

In this prospective study, we observed a dose–response association between prediagnostic systolic and diastolic BP and prostate cancer risk and overall survival. Additionally, among prostate cancer patients treated with curative intention and patients receiving endocrine treatment a high prediagnostic diastolic BP (≥100 mmHg) was associated with a threefold and twofold increased increased overall mortality risk, respectively, compared with those with a prediagnostic diastolic BP below 80 mmHg.

Our results extend previous results but are also in part supported by others who have observed that elevated systolic BP is associated with increased incidence of prostate cancer.^25^, ^26^, ^27^, ^28^ Interestingly, hypertension was associated with higher prostate cancer risk, with the strongest association for fatal prostate cancer.^12^ In contrast, neither the EPIC‐study nor a meta‐analysis observed any associations between hypertension and risk of prostate cancer.^7^, ^8^ Our findings that elevated prediagnostic systolic BP might be a risk factor only in men above 45 years may be an observation only by chance or may suggest variation by age groups and a reason for the inconsistent findings observed in previous studies. Of note, in a Swedish study including 330,000 men that were enrolled into the study between 1971 and 1993 with a mean age at entry of 34.7 years, both systolic and diastolic BP were associated with a decreased risk of incident prostate cancer.^29^ These findings may suggest that the association between elevated BP and prostate cancer may vary by time periods due to several factors, including improved diagnostic possibility of prostate cancer and an aging population at risk. Importantly, biological mechanism risk factors including chronic inflammation initiating raised systolic and diastolic BP may also vary throughout different time periods, and treatment for hypertension is initiated at a lower level of diastolic and systolic BP today compared with 1970s‐ ‘80s. These settings may complicate interpretation and comparisons between studies regarding raised BP and prostate cancer risk and survival throughout time periods, even if tracking of BP is high.^22^ Furthermore, the age at onset of hypertension and the cumulative exposure of hypertension during lifetime may complicate the interpretation of any association between elevated BP and prostate cancer during long‐term follow‐up. Of note, all our participants have measured BP at study entry.

Few studies have looked at the isolated effect of diastolic BP on prostate cancer development, but among patients with prostate cancer with a mean age at diagnosis of 70 years, high levels of PSA were associated with high levels of systolic and diastolic BP.^30^ In another study, a positive association between PSA and diastolic BP was observed when adjusting for age and other clinical and socioeconomic factors,^31^ and a 5% increased risk for prostate cancer for each 11.4 mmHg increase in prediagnostic diastolic BP has been observed by others.^32^ These findings support our findings suggesting that elevated diastolic BP may play a role in relation to prostate cancer development.

To our knowledge, we are the first to investigate the effect of prediagnostic diastolic BP by treatment details (curative intent, endocrine treatment). However, our findings of a threefold increased mortality risk among patients with prostate cancer receiving curative treatment with a prediagnostic diastolic BP >100 mmHg compared with patients with prostate cancer with diastolic BP <80 mmHg are in part supported. Moustsen et al. observed that men who received first‐line palliative treatment had higher rates of ischemic stroke or heart failure, compared with prostate cancer–free men.^33^ These findings are also in line with our observation that men with prostate cancer die at an earlier age than prostate cancer–free men (Table S2). In addition, in a retrospective cohort study with 1900 patients with nonmetastatic prostate cancer, 10 years after diagnosis the cumulative probability of prostate cancer mortality and CVD mortality was 16.4% and 10.0%, respectively.^34^ These findings support our findings as we observed that patients with prostate cancer died at an earlier age if they died due to prostate cancer than if they died of CVDs. Furthermore, pre‐existing hypertension, hyperglycemia, and overweight were associated with poor prostate cancer prognosis.^35^ Of note, in our study, diabetes and body composition were included as covariates in our final model, as they influenced our risk estimates.

Recently, cardiovascular health, including optimal BP, is suggested to be an important factor when balancing disease management and monitoring cardiovascular health in patients with prostate cancer. The importance of including optimal BP treatment among patients with prostate cancer was underlined in a recent study, as men who received first‐line palliative treatment had higher rates of heart failure and ischemic stroke.^33^

Systemic inflammation is among the potential biological mechanisms operating to explain the observed association between hypertension and prostate cancer.^36^, ^37^, ^38^ Inflammation is one of the hallmarks of prostate cancer development,^3^ and inflammatory cells associated with precursor lesions for prostate cancer in the prostate gland, have been observed.^4^ Interestingly, our results suggest that elevated diastolic BP is a stronger risk factor than elevated systolic BP for prostate cancer development, and in particular for mortality risk. Whether diastolic BP rather than systolic BP is more linked to chronic inflammation is not much studied.^39^ However, the main determinants of the systemic arterial BP is cardiac output, systemic vascular resistance, and a critical closing pressure at the level of the arterioles.^40^ Raised BP may downregulate IGF‐binding protein‐1 (IGFBP‐1), and this might increase the risk of prostate cancer by increasing IGF‐1 activity.^32^ More research is needed to determine whether systemic inflammation caused by both raised systolic and diastolic BP play a role or share common biological pathways influencing prostate cancer development, or if premalignant cells cause the inflammation that causes the hypertension.

The strengths of our study include the measured BP, its population based and prospective design with high attendance rate, and a high completeness rate of identification of patients with prostate cancer (98.8%).^41^ Furthermore, the rather long, follow‐up time, which may result in long exposure time of elevated BP, the broad information about baseline characteristics and precise measurements of risk factors strengthens the results observed. All medical records for the patients with prostate were carefully reviewed by trained physicians with systematic abstraction of histopathology and clinical characteristics. The study was able to control for several potential confounding factors, and to address effect modification, such as age, BMI, smoking habits, diabetes, and physical activity.

Our study also has some limitations. The exposure variables and other baseline variables were based on a single‐time, prediagnostic measure. However, tracking studies from the same cohort of men have shown that men tend to follow a trajectory of BP suggesting an accumulated lifetime exposure.^22^ The associations between all‐cause mortality and baseline BP among patients with prostate cancer (Table 3) are based on few events within each category, and results should be interpreted with care. The frequency of PSA‐testing in the population increased during the study period, which also influences the incidence of prostate cancer and the age at diagnosis.^42^ The year of prostate cancer diagnosis varies from 1996 to 2018 (median 2011). In the group aged <45 years at baseline (*n* = 161) the year of diagnosis varies from 1999 to 2018 (median 2015). In the group aged ≥45 at baseline (*n* = 650) the year of diagnosis varies from 1996 to 2018 (median 2010). The increase in PSA testing has been prominent regardless of age, and it seems less likely that this would affect our results^42^

The sample size was not large enough to conduct detailed subgroup analysis on the cause of death, and information regarding family history of prostate cancer was not available. We did not have access to serum testosterone levels at baseline and was not able to control for this factor in our analyze. Low testosterone concentrations may be an independent risk factor for hypertension in males.^43^, ^44^ Although ADT is a cornerstone in the treatment of metastatic prostate cancer, there is no solid evidence regarding the testosterone level and risk of prostate cancer,^45^ but testosterone levels might influence both BP and prostate cancer development and could be an important factor. We did not have access to genetic analyses, in particular polygenic hazard scores, which might be an up‐and‐coming tool for prostate cancer risk stratification.

In conclusion, our study supports that both elevated prediagnostic systolic and diastolic BP are associated with prostate risk, and with overall mortality among patients with prostate cancer. These findings underline that both systolic and diastolic BP are important factors when balancing disease management and monitoring cardiovascular health in patients with prostate cancer. Our results are based on a single data point of BP, and should be interpreted with caution, and further studies are needed. Nevertheless, the present study supports the view that clinical follow‐up visits of patients with prostate cancer should include measuring BP and initiate hypertensive treatment when appropriate, to balance and optimize the management of patients with prostate cancer.

## CONFLICTS OF INTEREST

The authors have declared no conflicts of interest.

## AUTHORS’ CONTRIBUTION

Conceived the study: ES, ER, HSH, and IT. Interpretation of data from the Tromsø study: TW, AEE, ES, ER, IT, HSH, and HS. Constructed the clinical database: ES, TK, MS, IT, HSH, and ER. Performed histological examination: ER. Performed statistical analyses and drafted the manuscript: ES, TW, ER, HSH, EG, AM, and IT. Critically reviewed the manuscript: all authors. Approved the final manuscript: all authors.

## ETHICS

This study has been approved by the Regional Committee for Medical and Health Research Ethics North (REK) (2015/1059) and was performed in accordance with the 1964 Helsinki Declaration and its later amendments. Informed consent was obtained from all individual participants included in the study.

## Supporting information

Fig S1

Table S1‐S2

## Data Availability

The data set used in our study is available upon request, pending permission from the Tromsø Study (www.tromsoundersokelsen.no).
