# Supplementary figures and images for: Systolic and diastolic blood pressure, prostate cancer risk, treatment, and survival. The PROCA‐life study
Source: Cancer Med. 2021 Dec 22;11(4):1005–15. doi: 10.1002/cam4.4523 (PMC8855905; doi:10.1002/cam4.4523)

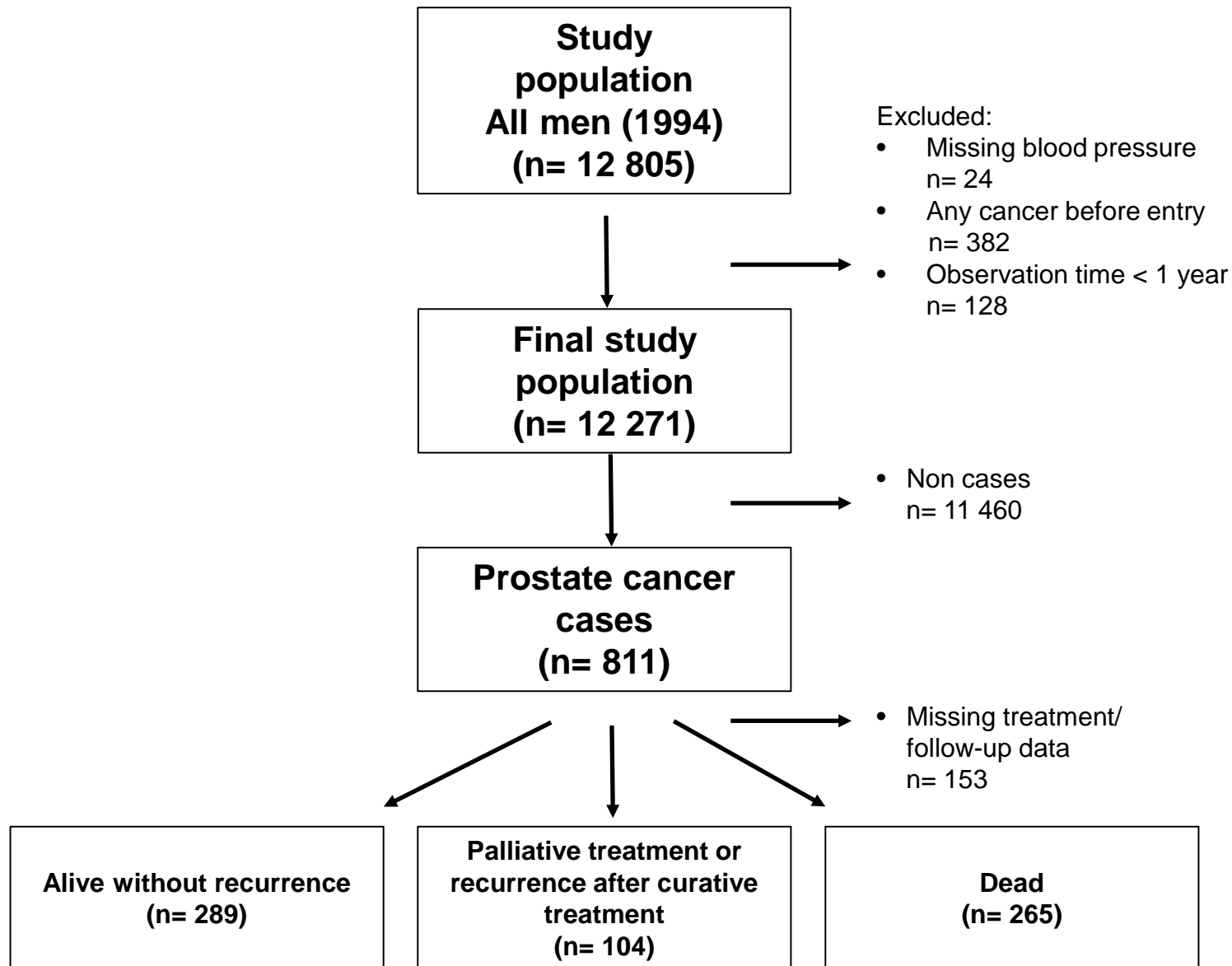

Supplement: Supplementary file 1 — Fig S1 [file CAM4-11-1005-s001.pdf]
