# Supplementary material for: Systolic and diastolic blood pressure, prostate cancer risk, treatment, and survival. The PROCA‐life study
Source: Cancer Med. 2021 Dec 22;11(4):1005–15. doi: 10.1002/cam4.4523 (PMC8855905; doi:10.1002/cam4.4523)
Supplement: Supplementary file 2 — Table S1‐S2 [file CAM4-11-1005-s002.doc]

**Supplementary results**

Supplementary table 1

Multivariable adjusted* hazard ratios (HRs) for mortality according to prediagnostic systolic and diastolic blood pressure among prostate cancer cases by age-group (**≤**/>45 years). The PROCA*-life* study (1994-2018).

|  |  | **Age at baseline**  **≤ 45 years** |  | **Age at baseline**  **> 45 years** |
| --- | --- | --- | --- | --- |
|  | Number of dead/ cases | 17/183 | Number of dead/ cases | 248/628 |
| **Systolic blood pressure (mmHg)** | |  |  |  |
| <130 | 10/95 | 1.00 (reference) | 57/201 | 1.00 (reference) |
| 130-139.9 | 5/56 | 0.92 (0.39-2.81) | 55/165 | 1.12 (0.76-1.64) |
| 140-149.9 | 2/23 | 1.44 (0.29-7.21) | 44/98 | 0.96 (0.62-1.45) |
| >=150 | 0/9 | n.a | 92/164 | 1.42 (1.00-2.02) |
| *P for trend†* |  | *0.84* |  | *0.06* |
| *Per SD increase* |  | *1.18 (0.53-2.60)* |  | ***1.15 (1.04-1.29)*** |
| **Diastolic blood pressure (mmHg)** | |  |  |  |
| <80 | 13/132 | 1.00 (reference) | 97/272 | 1.00 (reference) |
| 80-89.9 | 4/37 | 0.83 (0.24-2.84) | 71/190 | 1.14 (0.84-1.56) |
| 90-99.9 | 0/11 | n.a | 50/121 | 1.39 (0.97-1.98) |
| >=100 | 0/3 | n.a | 30/45 | **1.99 (1.30-3.04)** |
| *P for trend†* |  | *0.33* |  | ***0.002*** |
| *Per SD increase* |  | *0.74 (0.37-1.47)* |  | ***1.21 (1.07-1.37)*** |

*Adjusted for age at baseline, body mass index (bmi, kg/m^2^), smoking, alcohol use, physical activity, diabetes and education level

†P-value for linear trend in blood pressure categories.

Supplementary table 2

Mean age of death (years), and cause of death among prostate cancer cases during follow-up.

The PROCA*-life* study (1994-2018).

|  | **Prostate cancer cases** | |
| --- | --- | --- |
|  | N | Age of death (SD) |
| Prostate cancer death | 111 | 76.9 (10.8) |
| Cardiovascular death | 33 | 80.3 (7.6) |
| Other cause of death | 121 | 79.8 (7.3) |
| All death | 265 | 78.7 (9.1) |
